# Novel Hematologic Inflammatory Biomarker Ratios Derived From Routinely Ordered Labs Upon Admission: Enhancement of the International Mission for Prognosis and Analysis of Clinical Trials Prognostic Model for Moderate-to-Severe Traumatic Brain Injury

**DOI:** 10.7759/cureus.92601

**Published:** 2025-09-18

**Authors:** Kyle M Rei, Javed Siddiqi

**Affiliations:** 1 Neurology, University of Colorado Anschutz Medical Campus, Denver, USA; 2 Neurosurgery, Desert Regional Medical Center, Palm Springs, USA; 3 Neurosurgery, Riverside University Health System Medical Center, Moreno Valley, USA; 4 Neurosurgery, Arrowhead Regional Medical Center, Colton, USA; 5 Neurosurgery, California University of Science and Medicine, Colton, USA

**Keywords:** auroc, crash, hematologic inflammatory biomarkers, impact, moderate to severe tbi, mortality, neutrophil-to-lymphocyte ratio (nlr), prognostic model, traumatic brain injury (tbi), unfavorable outcomes

## Abstract

Introduction

Many novel hematologic inflammatory biomarker ratios derived from routinely ordered labs upon admission have demonstrated a significant predictive ability of six-month traumatic brain injury (TBI) outcomes. However, these biomarkers have not been evaluated in the context of existing validated TBI prognostic models.

Methods

This retrospective cohort study compared the predictive ability of 36 biomarkers measured upon admission to the International Mission for Prognosis and Analysis of Clinical Trials (IMPACT) prognostic model for six-month unfavorable outcomes and mortality of 199 neurosurgery patients between 2020 and 2024 in the United States. Model enhancement was evaluated via logistic regression comparing the IMPACT lab model to an extended model including one additional biomarker using likelihood ratio (LR) and Nagelkerke R².

Results

Of the 199 moderate-to-severe TBI patients included, 116 (58.3%) had six-month unfavorable outcomes, and 83 (41.7%) suffered six-month mortality. The IMPACT lab model showed excellent discrimination for both unfavorable outcomes (area under the receiver operating characteristic curve (AUROC) 0.887, p<.001) and mortality (AUROC 0.880, p<.001). Among 36 biomarkers, 19 showed significant discrimination on univariable analysis via AUROC. However, only 11 biomarkers remained significant after adjusting for the IMPACT lab model. Four biomarkers improved the IMPACT lab model for six-month unfavorable outcomes (R²=0.632): red cell distribution width (RDW) to platelet ratio (RPR) (LR=6.138, p=0.013, R²=0.654), lactate to platelet ratio (LPR) (LR=7.494, p=0.006, R²=0.672), prothrombin time (PT) (LR=5.636, p=0.018, R²=0.650), and lactate (LR=6.794, p=0.009, R²=0.670). Nine biomarkers improved the IMPACT lab model for six-month mortality (R²=0.659): RPR (LR=5.476, p=0.019, R²=0.678), LPR (LR=11.012, p<0.001, R²=0.694), lactate to albumin ratio (LAR) (LR=3.957, p=0.047, R²=0.737), platelets (LR=3.886, p=0.049, R²=0.672), lactate (LR=8.759, p=0.003, R²=0.686), albumin (LR=4.639, p=0.031, R²=0.739), PT (LR=12.764, p<0.001, R²=0.699), international normalized ratio (INR) (LR=12.02, p<0.001, R²=0.697), and activated partial thromboplastin time (aPTT) (LR=9.572, p=0.002, R²=0.688).

Conclusions

We demonstrated that many biomarkers showing significant predictive ability on univariable analysis lacked robustness when adjusting for a validated prognostic model, and only nine biomarkers enhanced the IMPACT lab model: RPR, LPR, LAR, platelets, PT, INR, aPTT, lactate, and albumin. RPR provided the IMPACT lab model with superior predictive ability for six-month unfavorable outcomes as compared to its individual constituent biomarkers, RDW and platelets, which demonstrates the utility of novel biomarker ratios. LPR and LAR did not provide the IMPACT lab model with superior predictive ability as compared to their constituent biomarkers, which may be preferred due to superior parsimony. Additional research is needed to validate the addition of these biomarkers to the IMPACT lab model. Future studies should evaluate TBI biomarkers in the context of existing validated models such as IMPACT or Corticosteroid Randomization After Significant Head Injury (CRASH).

## Introduction

Traumatic brain injury (TBI) is a major cause of death and disability worldwide [1]. In addition to primary mechanical injury, TBI is associated with secondary inflammation-mediated injury, which has been found to occur locally within the central nervous system (CNS) as well as systemically with widespread immune activation [2]. Local biomarkers may offer potential for future neuroprotective pharmacological interventions, while biomarkers of both local and systemic inflammation have demonstrated prognostic value [3].

Many studies have investigated the use of novel hematologic biomarker ratios derived from routinely ordered labs that are associated with systemic inflammation in predicting TBI outcomes: neutrophil-to-lymphocyte ratio (NLR) [4], systemic immune inflammation index (SII) [5], systemic inflammation response index (SIRI) [6], platelet-to-lymphocyte ratio (PLR) [7], monocyte-to-lymphocyte ratio (MLR) [8], segmented neutrophil-to-monocyte ratio (SeMo) [9], red cell distribution width (RDW)-to-platelet ratio (RPR) [10], hemoglobin-to-RDW ratio (HRR) [11], NLR times RPR (NLTRP) [12], prognostic nutritional index (PNI) [13], and lactate-to-albumin ratio (LAR) [14].

Similar biomarkers that have been investigated in the context of stroke, which has also been associated with systemic inflammation [15], to our knowledge, have not yet been investigated in the context of TBI: aggregate index of systemic inflammation (AISI) [16], platelet-to-white blood cell (WBC) ratio (PWR) [17], and albumin-to-globulin ratio (AGR) [18].

More broadly, other similar biomarkers associated with systemic inflammation that have been investigated in non-neurologic conditions include derived NLR (dNLR) for post-hoc clinical research as a surrogate for NLR where lymphocyte data may be unavailable [19]; platelet-to-monocyte ratio (PMR) for hepatitis B virus (HBV)-associated cirrhosis [20]; platelet-to-albumin ratio (PAR) for critically ill patients [21]; lactate-to-platelet ratio (LPR) for liver transplant [22]; and immature-to-total neutrophil ratio (I/T) for neonatal sepsis [23].

There are two notable externally validated TBI prognostic models derived from large multicenter studies with excellent predictive ability to discriminate between long-term unfavorable vs. favorable outcomes and mortality vs. survival (area under the receiver operating characteristic curves (AUROC) > 0.80): International Mission for Prognosis and Analysis of Clinical Trials (IMPACT) [24] and Corticosteroid Randomization After Significant Head injury (CRASH) [25]. Research investigating TBI biomarkers has been criticized for almost universally failing to adjust for these validated TBI prognostic models [26].

This study aims to retrospectively evaluate the predictive ability of 36 biomarkers (21 novel biomarker ratios vs. 15 individual constituent biomarkers; 13 novel biomarker ratios previously associated with TBI vs. eight novel biomarker ratios not yet investigated in the context of TBI) for six-month unfavorable vs. favorable outcomes and mortality vs. survival among moderate-to-severe TBI patients while adjusting for the IMPACT lab model and to evaluate whether these biomarkers can enhance the IMPACT lab model’s discrimination of these outcomes.

## Materials and methods

Participants

This single-center retrospective study included patients admitted to or consulted by the neurosurgery service at a level I trauma center located in the United States between June 2020 and June 2024. The inclusion criteria were as follows: (1) moderate-to-severe TBI (Glasgow Coma Scale (GCS) ≤ 12), as confirmed via history, clinical findings, and imaging at the time of initial evaluation by the neurosurgery service; (2) evaluated by the neurosurgery service within 24 hours of injury; and (3) at least 18 years of age. Exclusion criteria were as follows: (1) past medical history of stroke, neurosurgery, TBI, seizure, dementia, active anticoagulation, as well as neoplastic, cardiac, hepatic, and renal diseases; (2) TBI with penetrating mechanism; (3) loss to follow-up; and (4) missing data. Complete blood count (CBC) tests with manual differentials were treated as missing data and not considered in this analysis. This study was approved by the Institutional Review Board (IRB) of Arrowhead Regional Medical Center, Colton, CA (protocol #24-39).

Outcomes

Two outcomes were evaluated: (1) six-month unfavorable outcomes and (2) six-month mortality. Both outcomes were measured via the Glasgow Outcome Scale (GOS): GOS 1=death, GOS 2=vegetative state, GOS 3=severe disability (requiring assistance for activities of daily living (ADL)), GOS 4=moderate disability (some previous activities now no longer possible), and GOS 5=good recovery. Outcome 1 was dichotomized as GOS 1-3 (unfavorable) vs GOS 4-5 (favorable). Outcome 2 was dichotomized as GOS 1 (mortality) vs GOS 2-5 (survival) [27]. Similar to the methods used in the development of the IMPACT prognostic model, in patients whose six-month assessment was not available, we used the two-month OS (n=39, 19% of patients as compared to 19% in IMPACT) [24]. However, patients with major extracranial injuries (MEI) and less than six months of follow-up were treated as lost to follow-up and excluded due to foreseeable confounding.

Database review

The following admission clinical data were obtained: age, sex, injury mechanism, MEI, length of stay (LOS) in hospital, GCS total score, GCS motor score, pupillary reactivity, hypoxia, hypotension, follow-up period, and GOS. MEI was defined as an abbreviated injury scale (AIS) ≥ 3, or an injury requiring hospitalization on its own [28]. The following admission laboratory data was obtained: glucose (mmol/L), hemoglobin (Hgb, g/dL), WBC (103/uL), hematocrit (HCT, %), RDW (%), platelets (103/uL), neutrophil count (103/uL), lymphocyte count (103/uL), monocyte count (103/uL), immature granulocyte count (103/uL), albumin (g/dL), total protein (g/dL), globulin (g/dL), prothrombin time (PT, sec), international normalized ratio (INR), activated partial thromboplastin time (aPTT, sec), and lactate (mmol/L). Admission laboratory data were defined as the first lab value recorded upon admission, which was within 24 hours of injury. Admission laboratory data were not collected after a standardized time interval post-injury. The following admission imaging data were obtained: Marshall computed tomography (CT) classification, which includes midline shift (MLS), basal cistern compression/effacement, surgical evacuation of lesion, and presence of high/mixed density lesions > 25 cm³; traumatic subarachnoid hematoma (tSAH); and epidural hematoma (EDH).

IMPACT prognostic model

The IMPACT prognostic calculator was used to obtain the predicted risks associated with both outcomes using the IMPACT lab model (http://www.tbi-impact.org). The IMPACT lab model includes the following predictors: age, GCS motor score, pupil reactivity, hypoxia, hypotension, Marshall CT classification, tSAH, EDH, glucose, and Hgb [24].

Biomarkers

The following novel biomarker ratios were calculated as follows:

$${\displaylines{\text{NLR}=\frac{\text{Neutrophils}}{\text{Lymphocytes}}}}\tag{1}$$

$${\displaylines{\text{}dNLR=\frac{\text{Neutrophils}}{\text{WBC}-\text{Neutrophils}}}}\tag{2}$$

$${\displaylines{\text{SII}=\text{Platelets}\cdot \frac{\text{Neutrophils}}{\text{Lymphocytes}}}}\tag{3}$$

$${\displaylines{\text{SIRI}=\text{Monocytes}\cdot \frac{\text{Neutrophils}}{\text{Lymphocytes}}}}\tag{4}$$

$${\displaylines{\text{AISI}=\text{Platelets}\cdot \text{Monocytes}\cdot \frac{\text{Neutrophils}}{\text{Lymphocytes}}}}\tag{5}$$

$${\displaylines{\text{PLR}=\frac{\text{Platelets}}{\text{Lymphocytes}}}}\tag{6}$$

$${\displaylines{\text{MLR}=\frac{\text{Monocytes}}{\text{Lymphocytes}}}}\tag{7}$$

$${\displaylines{\text{SeMo}=\frac{\text{Segmented Neutrophils}}{\text{Monocytes}}}}\tag{8}$$

$${\displaylines{\text{Delayed SeMo}=\text{SeMo measured 48-72 hours after admission}}}\tag{9}$$

$${\displaylines{\text{Dynamic SeMo}=\text{Delayed SeMo} - \text{SeMo}}}\tag{10}$$

$${\displaylines{\text{RPR}=\frac{\text{RDW}}{\text{Platelets}}}}\tag{11}$$

$${\displaylines{\text{HRR}=\frac{\text{Hgb}}{\text{RDW}}}}\tag{12}$$

$${\displaylines{\text{NLTRP}=\frac{\text{Neutrophils}}{\text{Lymphocytes}}\cdot\frac{\text{RDW}}{\text{Platelets}}}}\tag{13}$$

$${\displaylines{\text{PMR}=\frac{\text{Platelets}}{\text{Monocytes}}}}\tag{14}$$

$${\displaylines{\text{PWR}=\frac{\text{Platelets}}{\text{WBC}}}}\tag{15}$$

$${\displaylines{\text{PNI}=(10 \cdot \text{Albumin}) + (0.005 \cdot \text{Lymphocytes})}}\tag{16}$$

$${\displaylines{\text{PAR}=\frac{\text{Platelets}}{\text{Albumin}}}}\tag{17}$$

$${\displaylines{\text{LPR}=\frac{\text{Lactate}}{\text{Platelets}}}}\tag{18}$$

$${\displaylines{\text{AGR}=\frac{\text{Albumin}}{\text{Globulin}}}}\tag{19}$$

$${\displaylines{\text{LAR}=\frac{\text{Lactate}}{\text{Albumin}}}}\tag{20}$$

$${\displaylines{\text{I/T}=\frac{\text{Immature Neutrophils}}{\text{Total Neutrophils}}}}\tag{21}$$

Statistical analyses

Statistical analysis was performed using IBM SPSS Statistics software, version 28.0.1.0 (142) (IBM Corp., Armonk, NY) and R version 4.3.1 (The R Core Team, R Foundation for Statistical Computing, Vienna, Austria) with the pROC and pwr packages. Skewed continuous data were analyzed via the Mann-Whitney test. Categorical data were analyzed via chi-square or Fisher's exact tests. AUROC was used to measure the ability of biomarkers to discriminate between patients with six-month unfavorable vs. favorable outcomes and six-month mortality vs survival. To evaluate whether biomarkers would provide additional prognostic value to the IMPACT lab model, a multivariable logistic regression model was created for each biomarker: outcome ~ IMPACT lab model predicted risk + biomarker [26]. A sensitivity analysis was then performed to also adjust for MEI: outcome ~ IMPACT lab model predicted risk + MEI + biomarker. Biomarkers found significant while adjusting for the IMPACT lab model were considered for further analysis. Multicollinearity was assessed using bivariate Pearson correlation coefficients. To evaluate the performance of adding these biomarkers to the IMPACT lab model, additional multivariable logistic regression models were created: outcome ~ age + GCS motor + pupil reactivity + hypoxia + hypotension + Marshall CT classification + SAH + EDH + glucose + hemoglobin + biomarker. Marshall CT classifications 5 and 6 were combined. Multicollinearity was again assessed using bivariate Pearson correlation coefficients. To internally validate the discrimination of these new models, five-fold cross-validation was performed. The median AUROC from each model applied to the test data was then compared to the AUROC from the IMPACT lab model-predicted risks (obtained via a free online calculator). DeLong’s test was used to compare these correlated AUROCs. Lastly, model enhancement was evaluated for each biomarker added to the IMPACT lab model via likelihood ratio (LR) and Nagelkerke R2. Effect size was measured via Cohen’s f². Post-hoc power calculation assumed alpha = 0.05 and power = 0.80. Statistical significance was defined as a p-value < 0.05.

## Results

Of the 199 patients included in this study, 116 (58.3%, 95% confidence interval (CI) 51.1%-64.9%) had an unfavorable outcome, and 83 (41.7%, 95% CI 35.1%-48.9%) suffered mortality within six months after sustaining a moderate-to-severe TBI. The median age was 29 years (IQR, 27 to 51 years), and 156 (78%) were male. The most common mechanism of injury was motor vehicle accident (MVA) (130, 65%), followed by falls (34, 17%) and other causes (35, 18%). The median GCS upon initial neurosurgery evaluation was seven (IQR, 3 to 10), with 82 (41%) patients sustaining moderate TBIs (GCS 9 to 12) and 117 (59%) patients sustaining severe TBIs (GCS 3 to 8). A minority of patients had MEI (89, 45%). A total of 45 (23%) patients underwent craniectomy for hematoma evacuation. Descriptive statistics for demographics and characteristics among all patients, those with unfavorable vs. favorable outcomes, and mortality vs. survival are described in Table 1. Descriptive statistics for hematologic biomarkers among all patients, those with unfavorable vs. favorable outcomes, and mortality vs. survival are described in Table 2.

**Table 1:** Difference in characteristics a: Mann-Whitney test; b: Chi-square or Fisher's exact test; Bolded p-values were significant (p<0.05) EDH: epidural hematoma; IMPACT: International Mission for Prognosis and Analysis of Clinical Trials; IQR: interquartile range; MVA: motor vehicle accident; tSAH: traumatic subarachnoid hemorrhage

		Outcome 1: Unfavorable Six-Month Outcome			Outcome 2: Mortality Six-Month Outcome		
Variables	All patients	Unfavorable	Favorable	p-value	Mortality	Survival	p-value
n	199	116	83		83	116	
Median age (IQR), years	29 (27-51)	39 (28-55)	36 (25-46)	0.034^a^	39 (28-64)	38 (26-47)	0.034^a^
Males, n (%)	156 (78)	90 (78)	66 (80)	0.744^b^	65 (78)	91 (78)	0.982^b^
Injury mechanism, n (%)				0.707^b^			0.520^b^
MVA	130 (65)	74 (64)	56 (68)		53 (64)	77 (66)	
Fall	34 (17)	22 (19)	12 (15)		17 (21)	17 (15)	
Other	35 (18)	20 (17)	15 (18)		13 (16)	22 (19)	
Major extracranial injury, n (%)	89 (45)	54 (47)	35 (42)	0.540^b^	36 (43)	53 (46)	0.746^b^
Median length of hospital stay (IQR), days	12 (3-27)	7 (1-27)	16 (8-28)	< .001^a^	3 (1-8)	22 (12-40)	< .001^a^
Median GCS total score (IQR)	7 (3-10)	4 (3-7)	10 (9-10)	< .001^a^	3 (3-7)	9 (7-10)	< .001^a^
TBI severity, n (%)				< .001^b^			< .001^b^
Moderate (GCS 9-12)	82 (41)	19 (16)	63 (76)		12 (15)	70 (60)	
Severe (GCS 3-8)	117 (59)	97 (84)	20 (24)		71 (86)	46 (40)	
GCS motor score, n (%)				< .001^b^			< .001^b^
None (1)	55 (28)	51 (44)	4 (5)		45 (54)	10 (9)	
Extension (2)	11 (6)	11 (10)	0 (0)		8 (10)	3 (3)	
Abnormal flexion (3)	8 (4)	6 (5)	2 (2)		4 (5)	4 (3)	
Normal flexion (4)	15 (8)	10 (9)	5 (6)		7 (8)	8 (7)	
Localizes/obeys (5/6)	110 (55)	38 (33)	72 (87)		19 (23)	91 (78)	
Pupillary reactivity, n (%)				< .001^b^			< .001^b^
Both pupils reacted	123 (62)	51 (44)	72 (87)		28 (34)	95 (82)	
One pupil reacted	19 (10)	11 (10)	8 (10)		6 (7)	13 (11)	
No pupil reacted	57 (29)	54 (47)	3 (4)		49 (59)	8 (7)	
Hypoxia, n (%)	23 (12)	15 (13)	8 (10)	0.474^b^	13 (16)	10 (9)	0.126^b^
Hypotension, n (%)	66 (33)	50 (43)	16 (19)	< .001^b^	39 (47)	27 (23)	< .001^b^
Marshall CT classification, n (%)				< .001^b^			< .001^b^
I	25 (13)	8 (7)	17 (21)		5 (6)	20 (17)	
II	83 (42)	32 (28)	51 (61)		19 (23)	64 (55)	
III	23 (12)	22 (19)	1 (1)		21 (25)	2 (2)	
IV	20 (10)	17 (15)	3 (4)		16 (19)	4 (3)	
V	45 (23)	34 (29)	11 (13)		19 (23)	26 (22)	
VI	3 (2)	3 (3)	0 (0)		3 (4)	0 (0)	
tSAH, n (%)	106 (53)	69 (60)	37 (45)	0.038^b^			0.168^b^
EDH, n (%)	21 (11)	13 (11)	8 (10)	0.723^b^			0.723^b^
Median IMPACT predicted unfavorable outcome risk at 6 months (IQR), %	40.1 (20.6-73.7)	65.2 (41.4-85.0)	19.5 (11.5-31.4)	< .001^a^	74.5 (52.3-87.4)	25.8 (13.2-39.5)	< .001^a^
Median IMPACT predicted mortality risk at 6 months (IQR), %	21.9 (10.4-51.1)	41.0 (22.8-65.1)	10.4 (6.2-16.2)	< .001^a^	51.9 (32.1-74.5)	13.3 (6.9-21.6)	< .001^a^

**Table 2:** Biochemical and hematological parameters upon admission a: Mann-Whitney test; Bolded p-values were significant (p<0.05) AISI/100: aggregate index of systemic inflammation divided by 100; AGR: albumin-to-globulin ratio; dNLR: derived neutrophil-to-lymphocyte ratio; HRR: hemoglobin-to-red cell distribution width (RDW) ratio; I/Tx100: immature-to-total neutrophil ratio multiplied by 100; LAR: lactate-to-albumin ratio; LPRx100: lactate-to-platelet ratio multiplied by 100; MLR: monocyte-to-lymphocyte ratio; NLR: neutrophil-to-lymphocyte ratio; NLTRP: neutrophil-to-lymphocyte ratio times RDW to platelet ratio; PAR: platelet-to-albumin ratio; PLR: platelet-to-lymphocyte ratio; PMR/100: platelet-to-monocyte ratio divided by 100; PNI: prognostic nutritional index; PWR: platelet-to-WBC ratio; RPRx100: RDW-to-platelet ratio multiplied by 100; SeMo: segmented neutrophil-to-monocyte ratio; SII/100: systemic immune inflammation index divided by 100; SIRI: systemic inflammation response index

			Outcome 1: Unfavorable Six-Month Outcome					Outcome 2: Mortality Six-Month Outcome				
Admission lab value	All patients		Unfavorable		Favorable		p-value^a^	Mortality		Survival		p-value^a^
	Median (IQR)	n	Median (IQR)	n	Median (IQR)	n		Median (IQR)	n	Median (IQR)	n	
Glucose, mmol/L	9.6 (7.4-12.8)	199	10.5 (8.5-15.5)	116	8.5 (7.0-10.0)	83	< .001	11.2 (8.9-16.1)	83	8.9 (7.0-10.4)	116	< .001
Hgb, g/dL	13.4 (11.8-14.5)	199	12.9 (11.4-14.2)	116	13.8 (12.3-14.7)	83	0.008	12.8 (10.8-14.3)	83	13.7 (12.3-14.6)	116	0.018
WBC, 10^3^/uL	14.3 (10.0-19.6)	199	14.4 (9.5-19.4)	116	14.3 (11.2-20.7)	83	0.361	13.4 (9.4-19.5)	83	15.0 (10.5-20.1)	116	0.125
HCT, %	40.3 (36.0-44.0)	199	39.0 (34.3-43.0)	116	41.6 (38.0-44.4)	83	0.003	39.0 (33.4-43.0)	83	41.2 (37.6-44.2)	116	0.008
RDW, %	13.0 (13.0-14.0)	199	13.0 (13.0-14.0)	116	13.0 (13.0-14.0)	83	0.056	13.0 (13.0-14.0)	83	13.0 (13.0-14.0)	116	0.253
Platelets, 10^3^/uL	242.0 (183.0-302.0)	199	216.0 (152.0-285.8)	116	278.0 (212.0-314.0)	83	< .001	199.0 (139.0-277.0)	83	270.0 (200.8-310.8)	116	< .001
Neutrophils, 10^3^/uL	7.9 (5.7-10.7)	123	8.3 (6.2-11.1)	73	7.7 (5.4-9.5)	50	0.113	8.0 (5.9-10.7)	49	7.9 (5.6-10.6)	74	0.897
Lymphocytes, 10^3^/uL	2.8 (1.9-4.3)	123	2.6 (1.7-4.2)	73	3.3 (2.2-4.4)	50	0.162	3.4 (1.7-4.3)	49	2.6 (2.1-4.3)	74	0.990
Monocytes, 10^3^/uL	0.7 (0.5-1.0)	123	0.7 (0.4-0.9)	73	0.7 (0.5-1.0)	50	0.306	0.6 (0.4-0.8)	49	0.7 (0.5-1.1)	74	0.021
Immature granulocytes, 10^3^/uL	0.1 (0.1-0.2)	123	0.1 (0.1-0.2)	73	0.1 (0.0-0.2)	50	0.198	0.1 (0.1-0.2)	49	0.1 (0.0-0.2)	74	0.315
Total neutrophils, 10^3^/uL	8.2 (5.9-11.1)	123	8.5 (6.5-11.5)	73	7.8 (5.6-9.6)	50	0.084	8.4 (5.9-10.9)	49	8.0 (5.8-11.2)	74	0.984
Albumin, g/dL	3.2 (2.7-3.9)	119	3.0 (2.5-3.7)	80	3.7 (3.1-4.2)	39	0.002	3.0 (2.5-3.5)	60	3.5 (3.0-4.2)	59	< .001
Total protein, g/dL	5.7 (4.9-6.8)	112	5.5 (4.8-6.5)	77	6.2 (5.6-7.1)	35	0.004	5.4 (4.7-6.2)	58	6.2 (5.6-7.0)	54	< .001
Globulin, g/dL	2.4 (2.0-2.8)	112	2.4 (1.8-2.8)	77	2.5 (2.3-2.9)	35	0.077	2.3 (1.7-2.7)	58	2.6 (2.3-2.9)	54	0.013
PT, sec	15.1 (13.9-17.1)	198	15.8 (14.3-18.6)	115	14.4 (13.5-15.8)	83	< .001	16.6 (14.9-19.9)	82	14.5 (13.6-15.8)	116	< .001
INR	1.2 (1.1-1.4)	198	1.3 (1.2-1.6)	115	1.2 (1.1-1.3)	83	< .001	1.4 (1.2-1.7)	82	1.2 (1.1-1.3)	116	< .001
aPTT, sec	30.1 (27.1-38.4)	198	33.0 (28.2-45.9)	115	28.9 (25.7-33.2)	83	< .001	36.6 (28.8-51.8)	82	28.9 (25.7-33.2)	116	< .001
Lactate, mmol/L	3.3 (1.7-5.2)	186	4.7 (2.3-6.4)	109	2.3 (1.5-3.5)	77	< .001	5.0 (2.8-6.9)	79	2.6 (1.5-4.0)	107	< .001
NLR	2.6 (1.6-5.3)	123	2.9 (1.6-5.6)	73	2.1 (1.2-4.5)	50	0.083	2.7 (1.6-5.3)	49	2.4 (1.4-5.3)	74	0.712
dNLR	1.9 (1.2-3.3)	123	2.1 (1.3-3.7)	73	1.6 (1.0-3.2)	50	0.082	2.0 (1.2-3.6)	49	1.8 (1.1-3.4)	74	0.547
SII/100	6.5 (3.4-11.8)	123	6.8 (3.2-12.3)	73	6.5 (3.4-11.1)	50	0.746	6.5 (2.7-11.4)	49	6.5 (3.7-11.9)	74	0.473
SIRI	1.7 (0.8-3.7)	123	1.7 (0.8-4.4)	73	1.5 (0.8-3.4)	50	0.455	1.5 (0.8-3.5)	49	2.1 (0.8-3.7)	74	0.250
AISI/100	4.2 (1.6-8.0)	123	4.3 (1.5-8.4)	73	4.2 (1.6-8.3)	50	0.861	3.3 (1.1-7.5)	49	4.9 (2.2-9.4)	74	0.100
PLR	80.3 (54.9-116.2)	123	78.3 (51.6-115.0)	73	81.3 (59.3-116.9)	50	0.404	70.0 (46.3-118.6)	49	85.2 (59.3-116.9)	74	0.112
MLR	0.2 (0.1-0.4)	123	0.2 (0.1-0.4)	73	0.2 (0.1-0.4)	50	0.823	0.2 (0.1-0.3)	49	0.3 (0.2-0.4)	74	0.096
SeMo	11.2 (7.6-16.0)	123	12.1 (8.8-18.0)	73	8.9 (7.5-12.7)	50	0.019	12.3 (9.4-20.9)	49	10.2 (7.5-13.2)	74	0.008
Delayed SeMo	12.1 (7.7-17.4)	86	13.8 (9.4-28.0)	52	9.2 (6.8-13.6)	34	0.011	13.6 (9.6-33.2)	33	10.1 (7.0-16.4)	53	0.025
Dynamic SeMo	1.0 (-3.4-6.3)	86	2.3 (-3.7-8.7)	52	-0.1 (-3.6-4.4)	34	0.273	2.5 (-4.6-14.2)	33	-0.1 (-3.3-5.4)	53	0.503
RPRx100	5.5 (4.4-7.5)	199	6.4 (4.6-9.2)	116	4.7 (4.2-6.0)	83	< .001	6.7 (4.8-10.4)	83	4.8 (4.3-6.8)	116	< .001
HRR	1.0 (0.9-1.1)	199	1.0 (0.8-1.1)	116	1.1 (0.9-1.2)	83	0.005	1.0 (0.8-1.1)	83	1.0 (0.9-1.1)	116	0.046
NLTRP	0.2 (0.1-0.4)	123	0.3 (0.1-0.4)	73	0.1 (0.1-0.2)	50	0.002	0.2 (0.1-0.4)	49	0.1 (0.1-0.4)	74	0.079
PMR/100	3.4 (2.4-4.8)	123	3.3 (2.1-4.8)	73	3.4 (2.7-5.0)	50	0.264	3.9 (2.5-5.1)	49	3.3 (2.4-4.6)	74	0.549
PWR	16.5 (11.9-21.6)	199	14.6 (11.2-20.2)	116	17.7 (14.2-23.1)	83	0.009	14.7 (11.2-20.0)	83	17.2 (13.0-23.1)	116	0.031
PNI	33.0 (27.0-39.0)	72	30.0 (27.0-38.0)	51	36.0 (31.5-40.5)	21	0.080	30.0 (27.0-37.0)	37	35.0 (27.0-41.0)	35	0.062
PAR	69.2 (55.3-92.3)	119	65.6 (52.8-91.7)	80	70.5 (56.7-95.5)	39	0.359	69.2 (46.5-94.4)	60	69.2 (56.8-88.3)	59	0.648
LPRx100	1.4 (0.6-2.6)	186	1.9 (0.9-3.6)	109	0.9 (0.5-1.4)	77	< .001	2.0 (1.0-4.6)	79	1.0 (0.5-1.6)	107	< .001
AGR	1.5 (1.2-1.7)	112	1.5 (1.2-1.7)	77	1.5 (1.3-1.7)	35	0.543	1.4 (1.2-1.7)	58	1.5 (1.2-1.7)	54	0.433
LAR	1.1 (0.6-2.0)	113	1.5 (0.7-2.6)	76	0.7 (0.4-1.1)	37	< .001	1.6 (0.8-3.0)	57	0.7 (0.4-1.4)	56	< .001
I/Tx100	1.6 (0.9-2.5)	123	1.6 (1.0-2.6)	73	1.6 (0.8-2.3)	50	0.660	1.7 (1.1-2.8)	49	1.6 (0.8-2.4)	74	0.494

The IMPACT lab model predicted risk obtained from the IMPACT prognostic calculator for six-month unfavorable outcome and mortality yielded excellent discrimination with AUROC 0.887 (95% CI 0.840-0.933, p<.001) and AUROC 0.880 (95% CI 0.832-0.928, p<.001), respectively (Table 3).

**Table 3:** AUROCs as compared to IMPACT predicted risks a: AUROC asymptotic significance; Bolded p-values were significant (p<0.05); IMPACT lab model six-month unfavorable outcome predicted risk: AUROC 0.887, 95% CI 0.840-0.933, p<.001; IMPACT lab model six-month mortality predicted risk: AUROC 0.880, 95% CI 0.832-0.928, p<.001. IMPACT: International Mission for Prognosis and Analysis of Clinical Trials; AUROC: area under the receiver operating characteristic curve; AISI/100: aggregate index of systemic inflammation divided by 100; AGR: albumin-to-globulin ratio; dNLR: derived neutrophil-to-lymphocyte ratio; HRR: hemoglobin-to-red cell distribution width (RDW) ratio; I/Tx100: immature-to-total neutrophil ratio multiplied by 100; LAR: lactate-to-albumin ratio; LPRx100: lactate-to-platelet ratio multiplied by 100; MLR: monocyte-to-lymphocyte ratio; NLR: neutrophil-to-lymphocyte ratio; NLTRP: neutrophil-to-lymphocyte ratio times RDW to platelet ratio; PAR: platelet-to-albumin ratio; PLR: platelet-to-lymphocyte ratio; PMR/100: platelet-to-monocyte ratio divided by 100; PNI: prognostic nutritional index; PWR: platelet-to-WBC ratio; RPRx100: RDW-to-platelet ratio multiplied by 100; SeMo: segmented neutrophil-to-monocyte ratio; SII/100: systemic immune inflammation index divided by 100; SIRI: systemic inflammation response index

	Outcome 1: Unfavorable Six-Month Outcome			Outcome 2: Mortality Six-Month Outcome		
Variables	n	AUROC (95% CI)	p-value^a^	n	AUROC (95% CI)	p-value^a^
Glucose, mmol/L	199	0.697 (0.624-0.770)	< .001	199	0.707 (0.632-0.781)	< .001
Hgb, g/dL	199	0.611 (0.533-0.689)	0.008	199	0.598 (0.516-0.681)	0.018
WBC, 10^3^/uL	199	0.462 (0.382-0.542)	0.361	199	0.436 (0.354-0.518)	0.125
HCT, %	199	0.624 (0.547-0.701)	0.003	199	0.610 (0.528-0.692)	0.008
RDW, %	199	0.576 (0.495-0.656)	0.069	199	0.545 (0.464-0.627)	0.276
Platelets, 10^3^/uL	199	0.683 (0.610-0.756)	< .001	199	0.678 (0.600-0.755)	< .001
Neutrophils, 10^3^/uL	123	0.584 (0.484-0.685)	0.113	123	0.507 (0.402-0.611)	0.897
Lymphocytes, 10^3^/uL	123	0.575 (0.472-0.677)	0.161	123	0.501 (0.395-0.607)	0.988
Monocytes, 10^3^/uL	123	0.554 (0.452-0.657)	0.307	123	0.623 (0.520-0.727)	0.021
Immature granulocytes, 10^3^/uL	123	0.568 (0.466-0.671)	0.198	123	0.554 (0.450-0.657)	0.315
Total neutrophils, 10^3^/uL	123	0.592 (0.492-0.692)	0.084	123	0.499 (0.395-0.603)	0.984
Albumin, g/dL	119	0.674 (0.570-0.778)	0.002	119	0.701 (0.606-0.795)	< .001
Total protein, g/dL	112	0.671 (0.565-0.778)	0.004	112	0.707 (0.610-0.803)	< .001
Globulin, g/dL	112	0.604 (0.496-0.713)	0.078	112	0.636 (0.533-0.739)	0.013
PT, sec	198	0.687 (0.615-0.760)	< .001	198	0.733 (0.660-0.807)	< .001
INR	198	0.679 (0.606-0.753)	< .001	198	0.729 (0.655-0.803)	< .001
aPTT, sec	198	0.691 (0.618-0.763)	< .001	198	0.732 (0.659-0.805)	< .001
Lactate, mmol/L	186	0.716 (0.643-0.790)	< .001	186	0.742 (0.669-0.815)	< .001
NLR	123	0.592 (0.490-0.694)	0.084	123	0.520 (0.415-0.624)	0.712
dNLR	123	0.594 (0.491-0.696)	0.078	123	0.531 (0.428-0.635)	0.558
SII/100	123	0.483 (0.380-0.585)	0.746	123	0.538 (0.432-0.645)	0.473
SIRI	123	0.540 (0.437-0.642)	0.455	123	0.439 (0.334-0.544)	0.251
AISI/100	123	0.509 (0.407-0.611)	0.861	123	0.588 (0.482-0.693)	0.100
PLR	123	0.544 (0.442-0.647)	0.404	123	0.585 (0.478-0.692)	0.112
MLR	123	0.511 (0.409-0.613)	0.837	123	0.411 (0.304-0.518)	0.095
SeMo	123	0.624 (0.524-0.724)	0.019	123	0.641 (0.539-0.743)	0.008
Delayed SeMo	86	0.663 (0.545-0.781)	0.011	86	0.644 (0.525-0.764)	0.025
Dynamic SeMo	86	0.570 (0.449-0.691)	0.273	86	0.543 (0.409-0.677)	0.503
RPRx100	199	0.685 (0.612-0.758)	< .001	199	0.675 (0.597-0.753)	< .001
HRR	199	0.618 (0.539-0.696)	0.005	199	0.583 (0.500-0.666)	0.046
NLTRP	123	0.664 (0.566-0.762)	0.002	123	0.594 (0.493-0.695)	0.079
PMR/100	123	0.559 (0.458-0.661)	0.264	123	0.468 (0.361-0.575)	0.549
PWR	199	0.609 (0.531-0.687)	0.009	199	0.590 (0.508-0.671)	0.031
PNI	72	0.632 (0.494-0.770)	0.080	72	0.628 (0.497-0.759)	0.062
PAR	119	0.552 (0.447-0.657)	0.359	119	0.524 (0.418-0.631)	0.648
LPRx100	186	0.741 (0.671-0.811)	< .001	186	0.757 (0.687-0.827)	< .001
AGR	112	0.536 (0.425-0.648)	0.538	112	0.544 (0.437-0.651)	0.425
LAR	113	0.744 (0.650-0.838)	< .001	113	0.745 (0.655-0.835)	< .001
I/Tx100	123	0.524 (0.419-0.628)	0.658	123	0.537 (0.433-0.641)	0.492

On univariable analysis, the following 18 biomarkers were associated with six-month unfavorable outcomes after moderate-to-severe TBI: glucose (AUROC 0.697, 95% CI 0.624-0.770, p<.001), Hgb (AUROC 0.611, 95% CI 0.533-0.689, p=0.008), HCT (AUROC 0.624, 95% CI 0.547-0.701, p=0.003), platelets (AUROC 0.683, 95% CI 0.610-0.756, p<.001), albumin (AUROC 0.674, 95% CI 0.570-0.778, p=0.002), total protein (AUROC 0.671, 95% CI 0.565-0.778, p=0.004), PT (AUROC 0.687, 95% CI 0.615-0.760, p<.001), INR (AUROC 0.679, 95% CI 0.606-0.753, p<.001), aPTT (AUROC 0.691, 95% CI 0.618-0.763, p<.001), Lactate (AUROC 0.716, 95% CI 0.643-0.790, p<.001), SeMo (AUROC 0.624, 95% CI 0.524-0.724, p=0.019), delayed SeMo (AUROC 0.663, 95% CI 0.545-0.781, p=0.011), RPR (AUROC 0.685, 95% CI 0.612-0.758, p<.001), HRR (AUROC 0.618, 95% CI 0.539-0.696, p=0.005), NLTRP (AUROC 0.664, 95% CI 0.566-0.762, p=0.002), PWR (AUROC 0.609, 95% CI 0.531-0.687, p=0.009), LPR (AUROC 0.741, 95% CI 0.671-0.811, p<.001), LAR (AUROC 0.744, 95% CI 0.650-0.838, p<.001) (Table 3).

On univariable analysis, the following 19 biomarkers were associated with six-month mortality after moderate-to-severe TBI: glucose (AUROC 0.707, 95% CI 0.632-0.781, p<.001), Hgb (AUROC 0.598, 95% CI 0.516-0.681, p=0.018), HCT (AUROC 0.610, 95% CI 0.528-0.692, p=0.008), platelets (AUROC 0.678, 95% CI 0.600-0.755, p<.001), monocytes (AUROC 0.623, 95% CI 0.520-0.727, p=0.021), albumin (AUROC 0.701, 95% CI 0.606-0.795, p<.001), total protein (AUROC 0.707, 95% CI 0.610-0.803, p<.001), globulin (AUROC 0.636, 95% CI 0.533-0.739, p=0.013), PT (AUROC 0.733, 95% CI 0.660-0.807, p<.001), INR (AUROC 0.729, 95% CI 0.655-0.803, p<.001), aPTT (AUROC 0.732, 95% CI 0.659-0.805, p<.001), lactate (AUROC 0.742, 95% CI 0.669-0.815, p<.001), SeMo (AUROC 0.641, 95% CI 0.539-0.743, p=0.008), delayed SeMo (AUROC 0.644, 95% CI 0.525-0.764, p=0.025), RPR (AUROC 0.675, 95% CI 0.597-0.753, p<.001), HRR (AUROC 0.583, 95% CI 0.500-0.666, p=0.046), PWR (AUROC 0.590, 95% CI 0.508-0.671, p=0.031), LPR (AUROC 0.757, 95% CI 0.687-0.827, p<.001), LAR (AUROC 0.745, 95% CI 0.655-0.835, p<.001) (Table 3).

On multivariable analysis, the following six biomarkers remained significantly associated with six-month unfavorable outcomes after adjusting for the IMPACT lab model predicted risks: platelets (odds ratio (OR) 0.993, 95% CI 0.988-0.998, p=0.006), PT (OR 1.204, 95% CI 1.003-1.446, p=0.047), lactate (OR 1.223, 95% CI 1.027-1.456, p=0.024), SIRI (OR 1.182, 95% CI 1.003-1.393, p=0.046), RPR (OR 1.275, 95% CI 1.058-1.536, p=0.011), and LPR (OR 1.434, 95% CI 1.031-1.995, p=0.032) (Table 4). Multicollinearity was not detected, with a maximum Pearson correlation coefficient < 0.5. In a sensitivity analysis, only PT became insignificant after also adjusting for MEI (OR 1.206, 95% CI 0.999-1.456, p=0.051).

**Table 4:** Logistic regression results for biomarkers predicting six-month outcomes after adjusting for IMPACT lab model predicted risks a: Logistic regression; Bolded p-values were significant (p<0.05). IMPACT lab model six-month unfavorable outcome: OR 1.075, 95% CI 1.054-1.097, p<.001. IMPACT lab model six-month mortality: OR 1.077, 95% CI 1.056-1.099, p<.001. Glucose and hemoglobin were not considered for this analysis as they are constituent variables of the IMPACT lab model. IMPACT: International Mission for Prognosis and Analysis of Clinical Trials; AUROC: area under the receiver operating characteristic curve; AISI/100: aggregate index of systemic inflammation divided by 100; AGR: albumin-to-globulin ratio; dNLR: derived neutrophil-to-lymphocyte ratio; HRR: hemoglobin-to-red cell distribution width (RDW) ratio; I/Tx100: immature-to-total neutrophil ratio multiplied by 100; LAR: lactate-to-albumin ratio; LPRx100: lactate-to-platelet ratio multiplied by 100; MLR: monocyte-to-lymphocyte ratio; NLR: neutrophil-to-lymphocyte ratio; NLTRP: neutrophil-to-lymphocyte ratio times RDW to platelet ratio; PAR: platelet-to-albumin ratio; PLR: platelet-to-lymphocyte ratio; PMR/100: platelet-to-monocyte ratio divided by 100; PNI: prognostic nutritional index; PWR: platelet-to-WBC ratio; RPRx100: RDW-to-platelet ratio multiplied by 100; SeMo: segmented neutrophil-to-monocyte ratio; SII/100: systemic immune inflammation index divided by 100; SIRI: systemic inflammation response index

		Outcome 1: Unfavorable Six-Month Outcome		Outcome 2: Mortality Six-Month Outcome	
Variables	n	OR (95% CI)	p-value^a^	OR (95% CI)	p-value^a^
WBC, 10^3^/uL	199	0.952 (0.900-1.007)	0.089	0.949 (0.898-1.002)	0.059
HCT, %	199	0.957 (0.898-1.019)	0.168	0.960 (0.905-1.019)	0.176
RDW, %	199	1.112 (0.883-1.402)	0.367	1.118 (0.908-1.377)	0.293
Platelets, 10^3^/uL	199	0.993 (0.988-0.998)	0.006	0.994 (0.989-0.999)	0.011
Neutrophils, 10^3^/uL	123	1.096 (0.949-1.267)	0.211	0.958 (0.837-1.098)	0.539
Lymphocytes, 10^3^/uL	123	0.747 (0.555-1.005)	0.054	0.827 (0.615-1.111)	0.207
Monocytes, 10^3^/uL	123	1.700 (0.466-6.197)	0.422	0.529 (0.153-1.826)	0.314
Immature granulocytes, 10^3^/uL	123	1.917 (0.526-6.988)	0.324	0.311 (0.011-8.977)	0.496
Total neutrophils, 10^3^/uL	123	1.119 (0.970-1.291)	0.124	0.954 (0.836-1.090)	0.492
Albumin, g/dL	119	0.599 (0.312-1.150)	0.124	0.428 (0.219-0.834)	0.013
Total protein, g/dL	112	0.730 (0.476-1.121)	0.150	0.623 (0.415-0.936)	0.023
Globulin, g/dL	112	0.736 (0.365-1.480)	0.389	0.677 (0.351-1.308)	0.246
PT, sec	198	1.204 (1.003-1.446)	0.047	1.243 (1.063-1.455)	0.007
INRx10	198	1.173 (0.993-1.384)	0.060	1.211 (1.049-1.398)	0.009
aPTT, sec	198	1.049 (0.997-1.103)	0.066	1.056 (1.014-1.100)	0.009
Lactate, mmol/L	186	1.223 (1.027-1.456)	0.024	1.238 (1.060-1.448)	0.007
NLR	123	1.143 (0.978-1.337)	0.093	1.055 (0.913-1.218)	0.469
dNLR	123	1.250 (0.928-1.684)	0.142	1.123 (0.834-1.512)	0.445
SII/100	123	1.048 (0.976-1.124)	0.196	1.009 (0.956-1.065)	0.735
SIRI	123	1.182 (1.003-1.393)	0.046	0.976 (0.845-1.126)	0.735
AISI/100	123	1.049 (0.986-1.115)	0.129	0.986 (0.933-1.043)	0.630
PLR	123	1.003 (0.996-1.010)	0.339	1.003 (0.998-1.009)	0.271
MLRx100	123	1.017 (0.998-1.037)	0.082	1.003 (0.986-1.020)	0.725
SeMo	123	0.994 (0.951-1.039)	0.786	1.012 (0.974-1.053)	0.535
Delayed SeMo	86	1.009 (0.974-1.046)	0.615	1.012 (0.983-1.042)	0.412
Dynamic SeMo	86	1.013 (0.980-1.047)	0.444	1.006 (0.979-1.033)	0.676
RPRx100	199	1.275 (1.058-1.536)	0.011	1.160 (1.034-1.302)	0.011
HRR	199	0.306 (0.049-1.894)	0.203	0.310 (0.053-1.813)	0.194
NLTRPx100	123	1.017 (0.998-1.037)	0.074	1.009 (0.993-1.025)	0.283
PMR/100	123	0.893 (0.750-1.063)	0.203	1.036 (0.882-1.217)	0.669
PWR	199	0.999 (0.957-1.042)	0.957	1.003 (0.962-1.047)	0.877
PNI	72	0.950 (0.868-1.040)	0.268	0.960 (0.881-1.046)	0.349
PAR	119	0.991 (0.975-1.008)	0.295	0.999 (0.983-1.015)	0.878
LPRx100	186	1.434 (1.031-1.995)	0.032	1.377 (1.089-1.741)	0.008
AGR	112	1.148 (0.308-4.274)	0.837	0.811 (0.234-2.812)	0.742
LAR	113	1.599 (0.920-2.779)	0.096	1.645 (1.060-2.554)	0.026
I/Tx100	123	1.058 (0.956-1.171)	0.272	0.894 (0.650-1.229)	0.490

On multivariable analysis, the following 10 biomarkers remained significantly associated with six-month mortality after adjusting for the IMPACT lab model predicted risks: platelets (OR 0.994, 95% CI 0.989-0.999, p=0.011), albumin (OR 0.428, 95% CI 0.219-0.834, p=0.013), total protein (OR 0.623, 95% CI 0.415-0.936, p=0.023), PT (OR 1.243, 95% CI 1.063-1.455, p=0.007), INR (OR 1.211, 95% CI 1.049-1.398, p=0.009), aPTT (OR 1.056, 95% CI 1.014-1.100, p=0.009), lactate (OR 1.238, 95% CI 1.060-1.448, p=0.007), RPR (OR 1.160, 95% CI 1.034-1.302, p=0.011), LPR (OR 1.377, 95% CI 1.089-1.741, p=0.008), LAR (OR 1.645, 95% CI 1.060-2.554, p=0.026) (Table 4). Multicollinearity was not detected, with a maximum Pearson correlation coefficient < 0.5. In a sensitivity analysis, none of the biomarkers became insignificant after adjusting for MEI.

Biomarkers found to be significant after adjusting for the IMPACT lab model were trained in new multivariable logistic regression models with the predictors from the IMPACT lab model using five-fold cross-validation. Comparing the median AUROC from these new models with the AUROC obtained from the IMPACT prognostic calculator, none showed a significant difference for either outcome via DeLong’s Test (Table 5 and Table 6). Large confidence intervals were observed due to small test data sample sizes ranging from n=22 to n=40. Multicollinearity was not detected, with a maximum Pearson correlation coefficient < 0.5.

**Table 5:** Six-month unfavorable outcome: comparison among five-fold cross-validation test data of median AUROC of IMPACT lab model plus additional variable and. AUROC of IMPACT lab model a: DeLong’s test IMPACT: International Mission for Prognosis and Analysis of Clinical Trials; AUROC: area under the receiver operating characteristic curve; LPRx100: lactate-to-platelet ratio multiplied by 100; RPRx100: red cell distribution width (RDW)-to-platelet ratio multiplied by 100; PT: prothrombin time; SIRI: systemic inflammation response index

Variables	n	Median AUROC of IMPACT Lab Model Plus Additional Variable (95% CI)	AUROC of IMPACT Lab Model (95% CI)	p-value^a^
Platelets	40	0.854 (0.732-0.977)	0.935 (0.863-0.999)	0.165
RPRx100	40	0.869 (0.757-0.981)	0.935 (0.863-0.999)	0.227
PT	40	0.867 (0.751-0.983)	0.860 (0.733-0.987)	0.771
Lactate	37	0.850 (0.718-0.982)	0.937 (0.863-0.999)	0.096
LPRx100	37	0.857 (0.727-0.987)	0.937 (0.863-0.999)	0.106
SIRI	25	0.801 (0.627-0.976)	0.917 (0.787-0.999)	0.164

**Table 6:** Six-month mortality: comparison among five-fold cross validation test data of median AUROC of IMPACT lab model plus additional variable and AUROC of IMPACT lab model a: DeLong’s test IMPACT: International Mission for Prognosis and Analysis of Clinical Trials; AUROC: area under the receiver operating characteristic curve; LAR: lactate-to-albumin ratio; LPRx100: lactate-to-platelet ratio multiplied by 100; RPRx100: red cell distribution width (RDW)-to-platelet ratio multiplied by 100; INRx100: international normalized ratio (INR) multiplied by 100; PT: prothrombin time; aPTT: activated partial thromboplastin time

Variables	Median AUROC of IMPACT Lab Model plus additional variable (95% CI)	AUROC of IMPACT Lab Model (95% CI)	p-value^a^
Platelets	0.871 (0.765-0.978)	0.891 (0.790-0.993)	0.707
RPRx100	0.881 (0.777-0.986)	0.891 (0.790-0.993)	0.847
PT	0.896 (0.797-0.996)	0.891 (0.790-0.993)	0.932
INRx100	0.896 (0.797-0.996)	0.891 (0.790-0.993)	0.932
aPTT	0.896 (0.795-0.998)	0.891 (0.790-0.993)	0.928
Lactate	0.869 (0.752-0.986)	0.872 (0.755-0.989)	0.961
LPRx100	0.896 (0.798-0.994)	0.872 (0.755-0.989)	0.704
Albumin	0.846 (0.674-0.999)	0.888 (0.730-0.999)	0.485
LAR	0.818 (0.619-0.999)	0.843 (0.673-0.999)	0.743
Protein	0.821 (0.636-0.999)	0.795 (0.602-0.988)	0.784

Model enhancement analysis revealed that the addition of the following four biomarkers enhanced the IMPACT lab model's ability to discriminate six-month unfavorable outcomes: RPR (LR 6.138, p=0.013, R²=0.654, f²=0.06), PT (LR 5.636, p=0.018, R²=0.650, f²=0.05), lactate (LR 6.794, p=0.009, R²=0.670, f²=0.12), and LPR (LR 7.494, p=0.006, R²=0.672, f²=0.12) (Table 7). For reference, post-hoc power calculations were provided with an alpha of 0.05, a power of 0.80, and Cohen’s f² as the effect size to determine the required sample size for detecting a significant model enhancement.

**Table 7:** Six-month unfavorable outcome: likelihood ratio analysis of IMPACT lab model a: Likelihood ratio test Bolded p-values were significant (p<0.05). IMPACT lab model Nagelkerke R^2 ^was 0.632. IMPACT: International Mission for Prognosis and Analysis of Clinical Trials; LPRx100: lactate to platelet ratio multiplied by 100; RPRx100: RDW to platelet ratio multiplied by 100.

Model	n	Likelihood Ratio	p Value^a^	Nagelkerke R^2^	Cohen's f^2^	Power Calculation (n)
IMPACT lab model + Platelets	199	3.774	0.052	0.645	0.04	199
IMPACT lab model + RPR	199	6.138	0.013	0.654	0.06	133
IMPACT lab model + PT	198	5.636	0.018	0.650	0.05	159
IMPACT lab model + Lactate	186	6.794	0.009	0.670	0.12	68
IMPACT lab model + LPR	186	7.494	0.006	0.672	0.12	68

Model enhancement analysis revealed that the addition of the following nine biomarkers enhanced the IMPACT lab model's ability to discriminate six-month mortality: platelets (LR 3.886, p=0.049, R²=0.672), RPR (LR 5.476, p=0.019, R²=0.678), PT (LR 12.764, p<0.001, R²=0.699), INR (LR 12.020, p<0.001, R²=0.697), aPTT (LR 9.572, p=0.002, R²=0.688), lactate (LR 8.759, p=0.003, R²=0.686), LPR (LR 11.012, p<0.001, R²=0.694), albumin (LR 4.639, p=0.031, R²=0.739), LAR (LR 3.957, p=0.047, R²=0.737) (Table 8). Similarly, for reference, power calculations were provided. 

**Table 8:** Six-month mortality: likelihood ratio analysis of IMPACT lab model a: Likelihood ratio test; Bolded p-values were significant (p<0.05). IMPACT lab model Nagelkerke R2 was 0.659. IMPACT: International Mission for Prognosis and Analysis of Clinical Trials; LAR: lactate-to-albumin ratio; LPRx100: lactate-to-platelet ratio multiplied by 100; RPRx100: red cell distribution width (RDW)-to-platelet ratio multiplied by 100; PT: prothrombin time; aPTT: activated partial thromboplastin time

Model	Likelihood Ratio	p-value^a^	Nagelkerke R^2^	Cohen's f^2^	Power Calculation (n)
IMPACT lab model + Platelets	3.886	0.049	0.672	0.04	199
IMPACT lab model + RPR	5.476	0.019	0.678	0.06	133
IMPACT lab model + PT	12.764	< .001	0.699	0.13	63
IMPACT lab model + INR	12.02	< .001	0.697	0.13	63
IMPACT lab model + aPTT	9.572	0.002	0.688	0.09	90
IMPACT lab model + Lactate	8.759	0.003	0.686	0.09	90
IMPACT lab model + LPR	11.012	< .001	0.694	0.11	74
IMPACT lab model + Albumin	4.639	0.031	0.739	0.31	28
IMPACT lab model + LAR	3.957	0.047	0.737	0.30	29
IMPACT lab model + Total Protein	3.224	0.073	0.722	0.23	37

**Figure 1:**
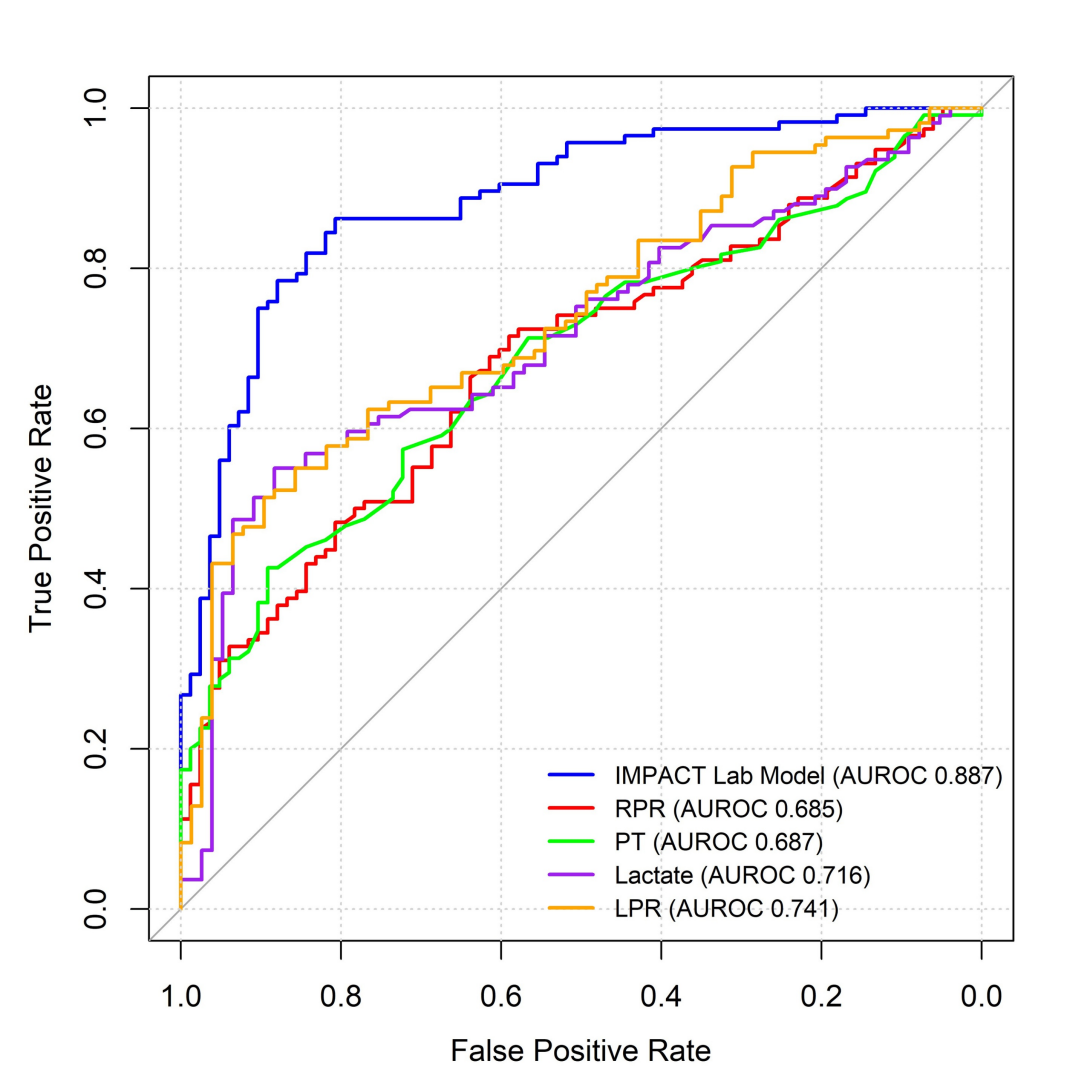
Univariable AUROC curves of the four admission hematologic biomarkers found to enhance the IMPACT lab model’s discrimination of six-month unfavorable vs. favorable outcomes after moderate-to-severe TBI, compared to the IMPACT lab model's predicted risk. IMPACT: International Mission for Prognosis and Analysis of Clinical Trials; AUROC: area under the receiver operating characteristic curve; PT: prothrombin time; LPR: lactate-to-platelet ratio; RPR: red cell distribution width (RDW)-to-platelet ratio.

**Figure 2:**
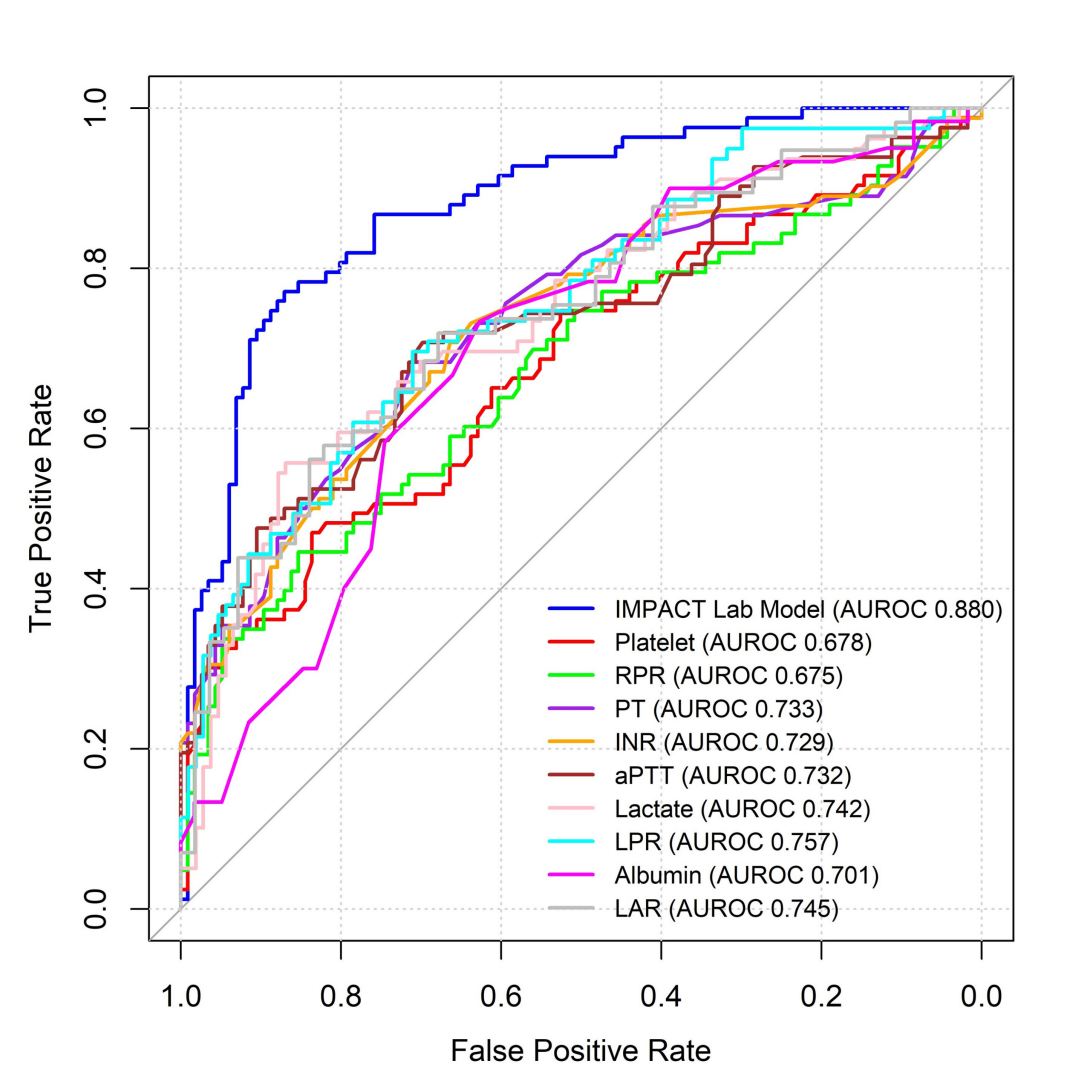
Univariable AUROC curves of the nine admission hematologic biomarkers found to enhance the IMPACT lab model’s discrimination of six-month mortality vs. survival after moderate-to-severe TBI compared to the IMPACT lab model predicted risk IMPACT: International Mission for Prognosis and Analysis of Clinical Trials; AUROC: area under the receiver operating characteristic curve; PT: prothrombin time; INR: international normalized ratio; aPTT: activated partial thromboplastin time; LPR: lactate-to-platelet ratio; RPR: red cell distribution width (RDW)-to-platelet ratio

Univariable AUROCs of admission hematologic biomarkers found to enhance the IMPACT lab model's ability to discriminate six-month unfavorable vs. favorable outcomes and six-month mortality vs. survival after moderate-to-severe TBI are presented in Figure 1 and Figure 2, respectively.

## Discussion

In total, 36 biomarkers (21 novel biomarker ratios vs. 15 individual constituent biomarkers; 13 novel biomarker ratios previously associated with TBI vs. eight novel biomarker ratios not yet investigated in the context of TBI) were investigated. Among these, 19 biomarkers showed significant discrimination on univariable analysis, which was inferior to the IMPACT lab model (AUROC 0.887 and 0.880 for six-month unfavorable outcome and mortality, respectively). After adjusting for the IMPACT lab model, 11 biomarkers remained significant, and nine biomarkers were shown to improve the IMPACT lab model. This study found that RPR, LPR, PT, and lactate improved the IMPACT lab model’s predictive ability of both six-month unfavorable outcomes and mortality. Additionally, LAR, platelets, INR, aPTT, and albumin improved the IMPACT lab model’s predictive ability of 6-month mortality only.

Among the 13 novel biomarker ratios previously found to be associated with TBI in the literature, only four (RPR, LPR, SIRI, and LAR) remained significant after adjusting for the IMPACT lab model. It has been suggested that biomarkers lacking this robustness may be surrogate markers for the severity of the underlying TBI, which are already explained in the IMPACT lab model via features like GCS motor score, pupil reactivity, midline shift, etc. [26]. This highlights the need to adjust for validated models and specifically test whether these models are enhanced by the addition of a biomarker. Five-fold cross-validation was attempted to quantify the improvement of model discrimination without overfitting by using the median AUROC with significance determined by comparison of the validated model via DeLong’s test; however, as confirmed in the post-hoc power calculation based on the model enhancement analysis, a larger sample size is needed to detect these small and medium effect sizes.

Among the final biomarkers shown to improve the IMPACT lab model, there were three novel biomarker ratios: RPR, LPR, and LAR. RPR was found to improve the IMPACT lab model for six-month unfavorable outcomes despite neither of its individual constituent biomarkers, RDW and platelets, improving the model’s detection of this outcome. This finding highlights the potential utility of novel biomarker ratios, which may offer superior predictive ability compared to their constituents.

Unlike RPR, LPR, and LAR, they failed to demonstrate superiority to their constituents, with lactate, platelets, and albumin also improving the IMPACT lab model. In this context, the inclusion of novel biomarker ratios may introduce unnecessary complexity to the model, and their constituents may be preferred due to their superior parsimony.

Possible mechanisms underlying these findings are described here. Post-TBI coagulopathy has been found to occur due to damaged brain parenchyma releasing tissue thromboplastin activating the extrinsic cascade, damaged endothelium directly activating platelets and the intrinsic cascade, and plasmin activation resulting in fibrinolysis [29]. Post-TBI RDW elevation likely reflects increased cytokines, such as tumor necrosis factor alpha (TNF-α), interleukin 1B (IL-1B), and IL-6, which inhibit erythropoietin-induced erythrocyte maturation and accelerate the release of larger reticulocytes [10]. While cerebral lactate metabolism has been shown to change after TBI via positron emission tomography (PET) and cerebral microdialysis (CMD) [30], serum hyperlactatemia post-TBI likely reflects the degree of tissue hypoperfusion and hypoxia [31], as well as hepatic and renal function [14], thereby functioning as a surrogate for injury severity. Albumin is a negative acute phase protein (APP), and its production is thought to decrease in the setting of inflammation, and by extension also post TBI, to conserve amino acids for positive APPs such as C-reactive protein, haptoglobin, angiotensinogen, etc. [32].

Lastly, we acknowledge the limitations of this study. First, being a single-center retrospective study makes it vulnerable to biases. Second, this study was likely underpowered to internally validate the enhanced models and quantify the magnitude of enhanced discrimination via AUROC. However, due to the paucity of literature on adjusting biomarkers for validated models, preliminary power calculations were not feasible. We present a post-hoc power calculation for future research. Third, the precise elapsed time from injury to biomarker measurement was not available, which introduced inherent heterogeneity. However, all biomarker measures were within 24 hours of injury. Fourth, similar to the IMPACT prognostic model, patients with MEI were included in this analysis. However, sensitivity analysis found that only one biomarker became insignificant after also adjusting for MEI (p=0.051). Fifth, similar to the IMPACT prognostic model, 19% of patients had an abbreviated time to follow up. However, patients with both MEI and abbreviated time to follow-up were excluded. Large prospective studies are needed to confirm the IMPACT prognostic model enhancement with the above biomarkers.

## Conclusions

We demonstrated that many biomarkers showing significant predictive ability on univariable analysis lacked robustness when adjusting for a validated prognostic model, and even fewer biomarkers improved the model. A methodology was presented to better evaluate biomarkers in the context of existing validated prognostic models to determine their additional prognostic value. The following four biomarkers improved the IMPACT lab model’s predictive ability of both six-month unfavorable outcomes and mortality: RPR, LPR, PT, and lactate. The following five biomarkers improved the IMPACT lab model’s predictive ability of six-month mortality only: LAR, platelets, INR, aPTT, and albumin. RPR provided the IMPACT lab model with superior predictive ability for six-month unfavorable outcomes as compared to its individual constituent biomarkers, RDW and platelets, which demonstrates the utility of novel biomarker ratios. LPR and LAR did not provide the IMPACT lab model with superior predictive ability as compared to their constituent biomarkers, which may be preferred due to superior parsimony. Additional prospective research is needed to validate the addition of these biomarkers to the IMPACT lab model. Future prospective studies should evaluate TBI biomarkers in the context of existing validated models such as IMPACT or CRASH.
